# Editorial: Interpersonal Wellbeing Across the Life Span

**DOI:** 10.3389/fpsyg.2022.840820

**Published:** 2022-02-11

**Authors:** Déborah Oliveira, Tim Carter, Aimee Aubeeluck

**Affiliations:** ^1^Division of Psychiatry, School of Medicine, Universidade Federal de São Paulo, São Paulo, Brazil; ^2^Faculty of Medicine, School of Health Sciences, University of Nottingham, Nottingham, United Kingdom

**Keywords:** interpersonal wellbeing, social wellbeing, lifespan, mental health, social connectedness, loneliness, social relationships

To most of us, healthy and nurturing interpersonal relationships are essential to our wellbeing. Such relationships can help us live longer, happier, and more meaningful lives. Conversely, where people sustain unhealthy relationships, they may experience long-term feelings of loneliness, alongside poor psychological and physical health, and may have higher rates of mortality. This Research Topic brings together seven influential articles from around the globe that consider how individual behaviors, thoughts, attitudes, emotions, and beliefs shape interpersonal experiences and influence wellbeing across the lifespan in different settings and considering different influencing factors, particularly among children and adolescents. Using a diverse range of qualitative and quantitative methodologies, these studies offer a broad insight into the wide range of interpersonal wellbeing research undertaken across the world, and in doing so, showcase the fundamental importance of our relationships with others and the interdependence of our behaviors, thoughts, attitudes, emotions, and beliefs and connection with our wellbeing, irrespective of country or setting.

Two studies shed light on interpersonal relationships and wellbeing among individuals accessing inpatient and outpatient mental health care services. Through an ethnographic study conducted in United Kingdom, Joyes et al. explored how social restrictions in institutionalized forensic care may affect interpersonal wellbeing among adults. The study showed how the inpatient mental health environment is complex and fraught with interpersonal issues related with trust, racism, the threat of physical violence and bullying, which were commonly experienced by staff and residents at the hospital. In Canada, Kealy et al. explored through a cross-sectional study using conditional process modeling the existing interaction between reduced emotional awareness and loneliness in 244 individuals attending community outpatient mental health care. The authors found an indirect effect of emotional awareness on loneliness which was mediated by distress concealment in mid-adulthood men, but not for women or older men, suggesting that interventions should focus on targeting restricted awareness and disclosure of emotional concerns to help young men to address the pain of loneliness. Both studies demonstrate that the link between social relationships and mental health care is strong and complex, regardless of the type of care setting or country, emphasizing the importance of involving interpersonal aspects and collaborative practices within mental health therapeutic models.

Three studies focusing on children and adolescents explored aspects related to early life development, violence, alcohol consumption in relation to loneliness and interpersonal relationships. In Europe, Hutten et al. examined the relationship between developmental patterns of loneliness and psychosocial functioning among 110 adolescents in the Netherlands using quantitative cluster analysis. The authors showed that higher levels of loneliness were present in adolescent groups who had anxiety, depression, and low self-esteem. The complexity of interpersonal relationships in adolescence was further explored by Cook et al. in the United Kingdom who showed how students who drink little or no alcohol may experience social exclusion on transition to University. Students described overt and covert pressure to drink and how this affected their ability to “fit in” with their peers. They note the importance of creating inclusive spaces and activities to improve student wellbeing.

Psychological resilience appears to be key to protect children and adolescents' mental health within multicultural families, and the quality of family dynamics and the mental health of teachers can have either a negative or a positive impact on the psychological wellbeing of young people. For example, a study conducted by Kim and Suh with multicultural children in South Korea revealed that hardiness and resilience were positively correlated with life satisfaction and life expectation, and negatively related with acculturative stress, and suggested that only when multicultural children had low resilience, the mediating effect of acculturative stress was significant in relations of hardiness and life satisfaction, as well as hardiness and life expectation. In another study, Li et al. tested a model on a sample of 1,100 adolescents in China and found that family violence predicted adolescent violence perpetration, and that this relationship was strongly mediated by peer association, normative beliefs, and negative emotions. Finally, in Australia, Carroll et al. used correlational and multi-level mediation analyses to find evidence of knock-on psychological benefits for students (*n* = 226) where teachers (*n* = 17) were provided with a 8-week stress-reduction intervention. Reductions in teachers' self-reported distress and burnout improved students' perceptions of their teachers' support and correlated with greater increases of academic self-perception in students.

It is clear from these papers how family, social, professional, and institutional relationships can impact on interpersonal wellbeing and this Research Topic provides insight into ways to tackle loneliness and social isolation that can so often be a product of poor relationships. This collection has highlighted the need to increase interpersonal wellbeing on a large scale and the importance of scientific evidence in understanding where our focus should be.

The studies presented furthers our understanding of interpersonal relationships (or interdependence) as a key determinant of wellbeing among children, adolescents, and communities across the world. None of the papers explored the key aspects related to interpersonal wellbeing among older people and we hope that other Research Topics can contribute to this important field of research among this population.

## Author Contributions

DO, TC, and AA were responsible for editing this Research Topic and drafting the manuscript. All authors contributed to the article and approved the submitted version.

## Conflict of Interest

The authors declare that the research was conducted in the absence of any commercial or financial relationships that could be construed as a potential conflict of interest.

## Publisher's Note

All claims expressed in this article are solely those of the authors and do not necessarily represent those of their affiliated organizations, or those of the publisher, the editors and the reviewers. Any product that may be evaluated in this article, or claim that may be made by its manufacturer, is not guaranteed or endorsed by the publisher.

